# Tolicizumab: a new option for refractory idiopathic recurrent pericarditis: a case report

**DOI:** 10.1093/ehjcr/ytaf535

**Published:** 2025-10-24

**Authors:** Valentino Collini, Francesco Venturelli, Filippo Quinzani, Roberta Sappa, Massimo Imazio

**Affiliations:** Cardiothoracic Department, University Hospital Santa Maria Della Misericordia, ASUFC, Piazzale Santa Maria della Misericordia 15, 33100 Udine, Italy; Cardiothoracic Department, University Hospital Santa Maria Della Misericordia, ASUFC, Piazzale Santa Maria della Misericordia 15, 33100 Udine, Italy; Department of Cardiovascular Department, Azienda Sanitaria Universitaria Giuliano Isontina (ASUGI), University of Trieste, Trieste, Italy; Division of Cardiology, Hospital of Cremona, Cremona, Italy; Cardiothoracic Department, University Hospital Santa Maria Della Misericordia, ASUFC, Piazzale Santa Maria della Misericordia 15, 33100 Udine, Italy; Cardiothoracic Department, University Hospital Santa Maria Della Misericordia, ASUFC, Piazzale Santa Maria della Misericordia 15, 33100 Udine, Italy; Department of Medicine (DMED), University of Udine, Udine, Italy

**Keywords:** Recurrent Pericarditis, Tocilizumab, IL-6 inhibitor, Case Report

## Abstract

**Background:**

Recurrent pericarditis is the most common and troublesome complication of pericarditis and can be particularly challenging when it becomes refractory to conventional therapies, often resulting in a prolonged disease course. Interleukin-1 (IL-1) inhibitors have transformed the management of such cases; however, not all patients tolerate or respond adequately to these agents.

**Case summary:**

We present a 37-year-old man suffering from idiopathic recurrent pericarditis since the age of 25. After failure of standard therapies, he became corticosteroid-dependent, so treatment with anakinra (an IL-1 inhibitor) was attempted twice, but discontinued early due to allergic reactions. As the recurrences were always characterised by an overt inflammatory phenotype (characterised by fever, increased C-reactive protein, and pericardial effusion), we administered off-label tocilizumab (an IL-6 receptor antagonist) as a last treatment option. The patient gained rapid benefit and was able to discontinue steroid therapy during the 6-month follow-up, achieving complete recovery from pericarditis.

**Discussion:**

Interleukin-6 is a key cytokine in the pathogenesis of pericarditis, acting synergistically with IL-1 to drive inflammation and acute phase responses. In our patient, blockade of IL-6 with tocilizumab led to the complete resolution of inflammation, suggesting a possible new therapeutic option for refractory cases. This result highlights the potential of IL-6 inhibition as an alternative strategy, when IL-1 blockers are contraindicated or ineffective.

Learning points

- Recurrent pericarditis can become resistant to NSAIDs, colchicine, and corticosteroids, requiring escalation to immunomodulatory therapies.
- IL-1 inhibitors are effective in many cases, but intolerance or inadequate response may occur and creates a therapeutic challenge calling for alternative strategies.
- IL-6 inhibition with tocilizumab could be a potential emerging therapy in selected patients when IL-1 blockers are contraindicated or ineffective.
- This is the first case report of a case of idiopathic recurrent pericarditis treated with such an approach due to the lack of therapeutic alternatives.

## Introduction

In recent decades, there has been a growing interest in pericardial diseases, and a new evidence-based approach to the treatment of acute and recurrent pericarditis (RP) has been introduced.^1,2^

The mainstay of medical therapy for the first episode of acute pericarditis (AP) is the empirical anti-inflammatory combination therapy with aspirin or nonsteroidal anti-inflammatory drugs (NSAIDs) plus colchicine.^1–3^ In case of contraindication of these drugs, or specific indications, corticosteroids (CS) should be considered at low to moderate doses plus colchicine.^4^ Despite these treatments, RP remains the most troublesome complication after a first attack of AP, affecting one-third of patients after the initial episode and up to 50% of patients with recurrences.^3–5^ In patients with multiple recurrences, triple therapy with NSAIDs, colchicine, and CS (tapered very slowly and eventually with IV route) may be considered, and for the most difficult cases with evidence of elevated C-reactive protein (CRP), recent clinical trials have demonstrated the effectiveness of anti-interleukin-1 (IL-1) drugs in dramatically reducing recurrences.^6–9^ However, when anti-IL1 drugs are contraindicated or ineffective, alternative therapies lack strong evidence-based clinical data.^10–12^ In this scenario, we administered Tocilizumab (a monoclonal antibody acting against the IL-6 receptor, widely used in inflammatory rheumatological diseases)^13–15^ to a male patient with recurrent idiopathic pericarditis refractory to conventional therapy.

## Summary figure

Timeline of the patient’s pericarditis course, key interventions, and outcomes. Initial episodes were managed with standard therapy (NSAIDs, colchicine, steroids) over several years. After failure of anakinra (due to severe adverse reaction) and IVIG, tocilizumab was introduced, coinciding with sustained remission.

**Figure:**
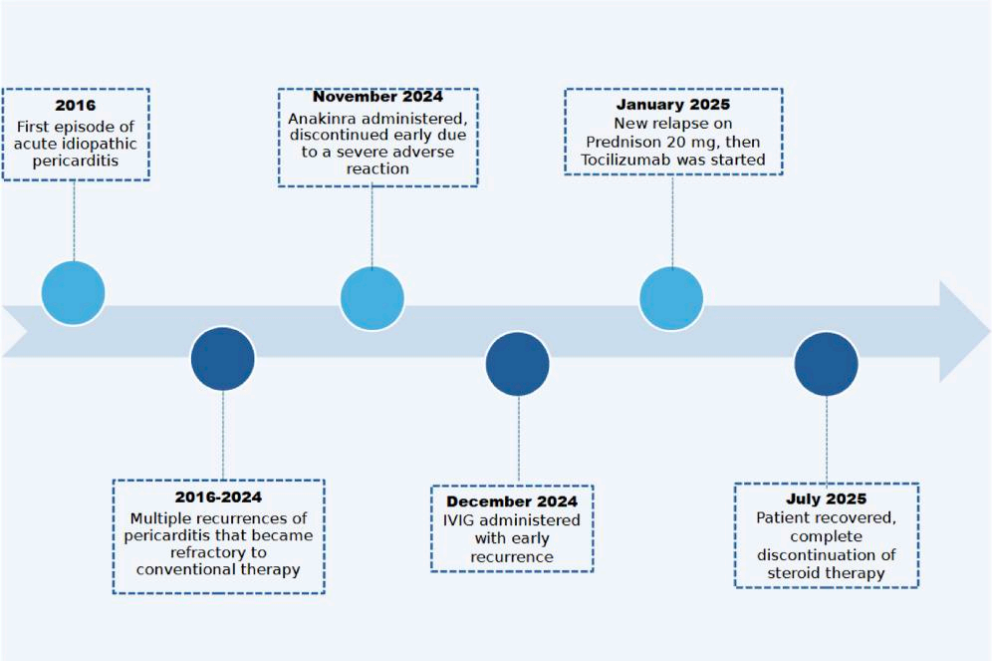


## Case presentation

**Figure 1:**
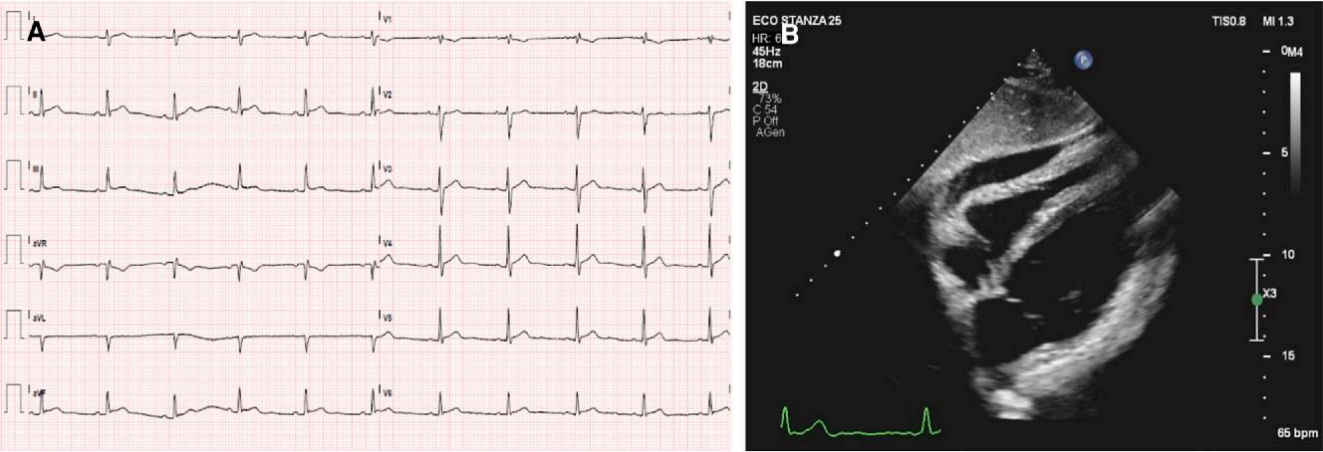
(*A*) the ECG demonstrated mild PR-segment downslope and ST-segment elevation in the inferior and lateral leads; (*B*) moderate pericardial effusion on echocardiography (subxiphoid view).

We report the case of a 37-year-old man who presented with his first episode of idiopathic acute pericarditis in 2016 at the age of 25, manifesting with pericardial chest pain, elevated fever, elevated markers of inflammation, and pericardial effusion (see *Figure 1*). The electrocardiogram (ECG) demonstrated mild PR-segment downslope and ST-segment elevation in the inferior and lateral leads (see *Figure 1*), while indices of myocardial necrosis were negative. He was treated with high-dose aspirin and colchicine, with resolution of the acute episode.

In the following years (2016–2024), the patient experienced multiple recurrences of pericarditis, managed with various combinations of NSAIDs, colchicine, and intermittent courses of low-dose corticosteroids. However, the patient became corticosteroid-dependent (experiencing recurrences of pain whenever prednisone was tapered); therefore, Anakinra was started, but discontinued early due to widespread allergic reaction.

In 2024, unfortunately, the patient had multiple recurrences, with symptoms partially improved with therapy, and recurred when steroids were tapered.

**Figure 2:**
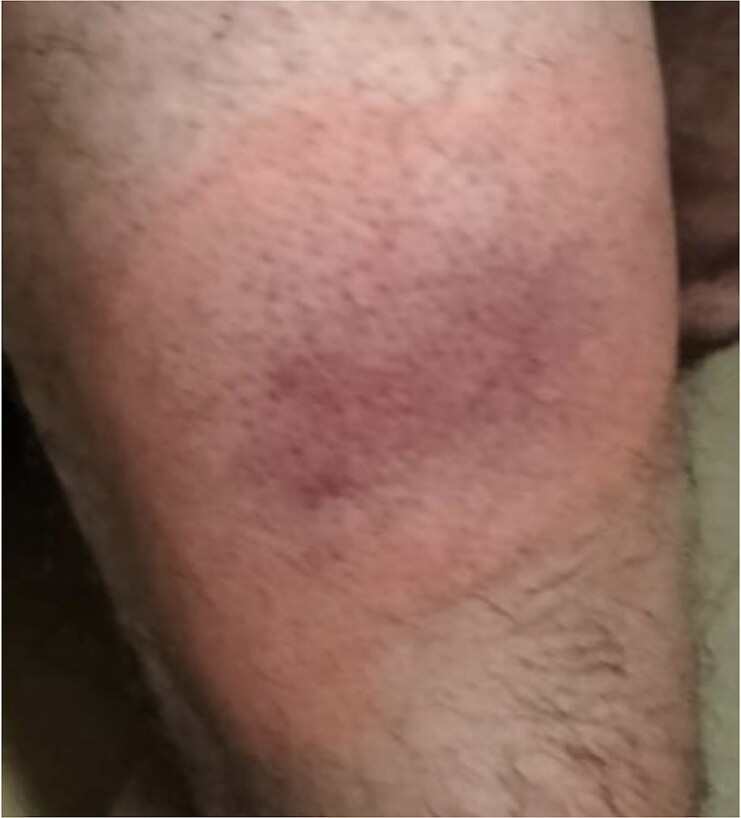
Severe adverse reactions at the injection site.

Following another severe relapse, anakinra was re-administered, but the patient developed another severe allergic reaction with erythematous foci at the injection site and generalized urticarial skin lesions (see *Figure 2*), leading to an immediate discontinuation of the drug.

Since IL-1 blockade was not feasible, intravenous immunoglobulin (IVIG) (a 5-day course of 400 mg/kg/day) was administered as immunomodulatory treatment. The treatment was well tolerated, but a few months later, while being treated with 20 mg/die of prednisone, the patient had another major relapse characterized by high fever, severe chest pain, large pericardial effusion, marked elevation of inflammatory markers (CRP 104 mg/L), and an episode of atrial fibrillation, cardioverted to sinus rhythm with IV flecainide.

Then he was admitted and treated with intravenous methylprednisolone and indomethacin, while continuing colchicine. At this point, given the refractory disease course and having relapsed on triple anti-inflammatory therapy, we initiated treatment with tocilizumab 162 mg SC weekly. A few weeks after starting therapy with tocilizumab, the patient reported a complete resolution of symptoms and returned to full activity. Corticosteroids were tapered off. An echocardiogram at 3 months post-tocilizumab showed no pericardial effusion; 6 months later, he remained symptom-free, inflammatory markers were normal, and he has had no further pericarditis recurrences.

## Discussion

**Figure 3:**
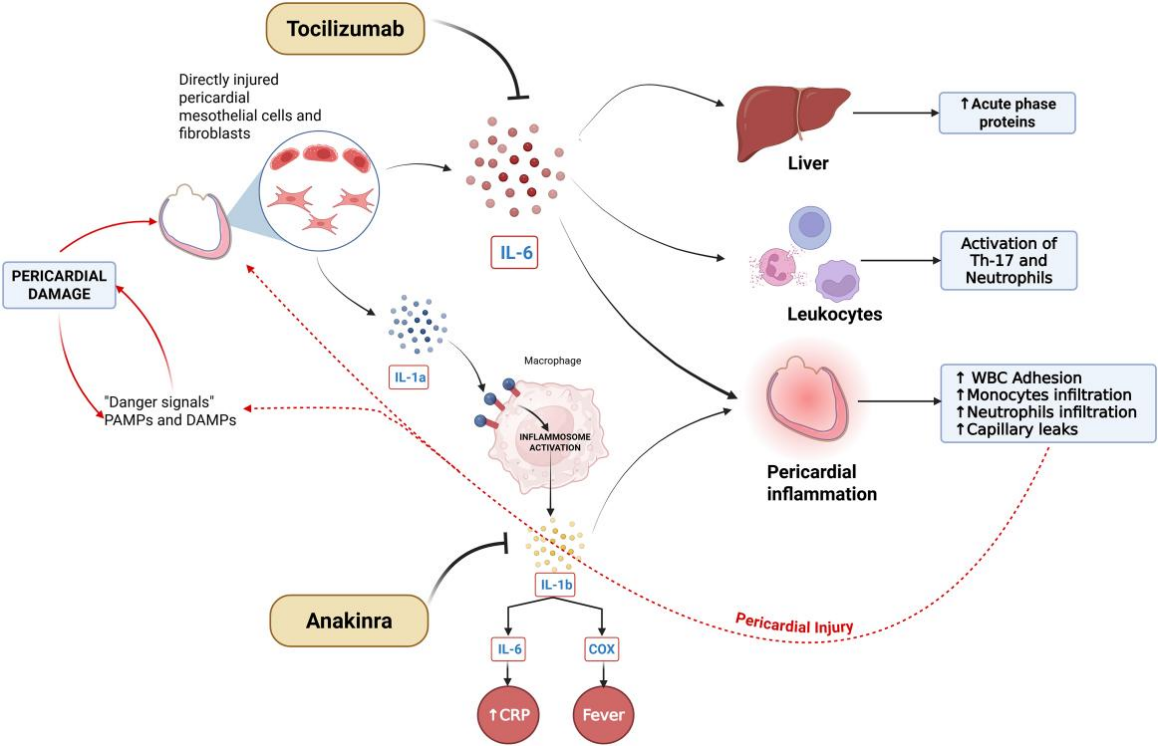
Interleukin-6 (IL-6) signalling via the membrane-bound receptor (classical signalling) and the soluble receptor (*trans*-signalling), both leading to downstream activation of inflammatory pathways. Tocilizumab, a monoclonal antibody targeting the IL-6 receptor, inhibits both signalling routes by preventing IL-6 binding and blocking the propagation of the inflammatory response.

To the best of our knowledge, this is the first case report illustrating the potential role of IL-6 inhibition in treating idiopathic recurrent corticosteroid-dependent colchicine-resistant pericarditis. The successful outcome in our patient aligns with the known pathophysiological importance of IL-6 in inflammatory processes.^13^ IL-6 is a pro-inflammatory cytokine that influences both innate and adaptive immunity, acting synergistically with IL-1 in driving the acute phase response (including induction of CRP and other inflammatory mediators). Indeed, interleukin-1 (IL-1) is an upstream cytokine that initiates the release of IL-6, whereas IL-6 acts downstream, amplifying and sustaining inflammation.^13^ By blocking IL-6 receptor signalling, tocilizumab disrupts pericardial inflammation and reduces CRP production, limiting inflammatory cell recruitment and modulating T-cell responses (*Figure 3*).^14^

In recent years, IL-1 inhibitors have revolutionized therapy for recurrent pericarditis refractory to conventional treatment.^6–9^ However, a subset of patients cannot tolerate IL-1 agents (as in our case) or may show persistent disease despite IL-1 blockade. Historically, such patients have been treated with IVIG or classic immunosuppressants (e.g. azathioprine), but robust evidence is lacking.^10–12^ Given IL-6’s role in the inflammatory cascade, IL-6 blockade represents a logical alternative target (*Table 1*).

**Table 1:** Comparison of anakinra, rilonacept, and tocilizumab for pericarditis

Drug	Mechanism of action	Dosing	Timing	Duration of therapy	Side effects	Monitoring	Costs (EUR)^a^
Anakinra	IL-1 receptor antagonist; blocks IL-1α and IL-1β activity	100 mg/day subcutaneous injection	Rapid onset, usually within days	Months; tapering based on clinical response	Injection site reactions, infections, neutropenia	Complete blood count (CBC), liver function, signs of infection	Approx. €1000–€1500/month
Rilonacept	IL-1 trap; binds IL-1α and IL-1β preventing receptor activation	320 mg loading dose, then 160 mg weekly SC	Rapid onset, often within days	Months; duration guided by recurrence risk	Injection site reactions, upper respiratory infections	CBC, lipid panel, signs of infection	Approx. €4000–€5000/month
Tocilizumab	IL-6 receptor antagonist	162 mg SC weekly	Slower onset, days to weeks	Variable; often prolonged in chronic cases	Infections, elevated liver enzymes, gastrointestinal side effects	CBC, liver function, lipids, infection markers	Approx. €1200–€1800/month

^a^Approximative costs in euros, they can vary in different EU countries; rilonacept is usually not available in EU countries.

Notably, IL-6 inhibition has shown benefit in other inflammatory conditions with serositis, such as adult-onset Still’s disease and systemic juvenile arthritis.^15–18^ Case reports have also described improvement in rheumatologic-associated pericarditis with tocilizumab.^17,18^ Our case adds novel evidence in idiopathic recurrent pericarditis. Compared with IL-1 inhibitors, IL-6 blockade tends to exert a slower but steady effect on inflammation, making gradual tapering of conventional anti-inflammatory therapy advisable. Safety profiles also differ: Anakinra often causes local injection reactions, while tocilizumab may induce liver enzyme elevations, lipid alterations, and increase infection risk. Careful monitoring of hepatic function, lipid profile, and infection markers is therefore recommended during therapy. Importantly, our patient did not experience any adverse effects throughout follow-up.

In summary, our case demonstrates that tocilizumab can induce remission in refractory recurrent pericarditis, offering new hope for patients with limited treatment options. This highlights the need for tailored immunotherapy guided by the predominant cytokine pathways driving individual disease.

## Data Availability

The data underlying this article cannot be shared publicly due to *the privacy of individual that participated in the case report.* The data will be shared on reasonable request to the corresponding author.

## Lead author biography

Dr Valentino Collini is a cardiology specialist working in close collaboration with Professor Massimo Imazio at Santa Maria della Misericordia University Hospital in Udine, Italy. He has a strong clinical and research interest in pericardial diseases and inflammatory cardiomyopathies, and is also a member of the ESC Working Group on Myocardial and Pericardial Diseases.

**Consent:** The authors confirm that written informed consent was obtained from the patient for publication of this case report and the accompanying images. The patient consented to the use of his medical information in accordance with COPE guidelines and institutional policies.

**Funding:** None.
